# Improved detection and quantification of peritoneal metastases using delayed contrast-enhanced dual-energy CT scans

**DOI:** 10.1186/s41747-025-00627-5

**Published:** 2025-10-08

**Authors:** Giulio Bagnacci, Armando Perrella, Nunzia Di Meglio, Vito Di Martino, Letizia Sansotta, Francesco Gentili, Susanna Guerrini, Silvia Ruggeri, Cristina Intrieri, Stefania Piccioni, Daniele Marrelli, Maria Antonietta Mazzei

**Affiliations:** 1 Department of Medical, Surgical and Neuro Sciences, University of Siena, Siena, Italy; 2 Unit of Diagnostic Imaging, Azienda Ospedaliero Universitaria Senese (AOUS), Siena, Italy; 3 Unit of Radiology, Azienda USL Toscana Nord Ovest, Pontedera, Pisa, Italy; 4 Department of Radiology, Azienda Ospedaliero Universitaria Careggi, Florence, Italy; 5 Surgical Oncology Unit, Azienda Ospedaliero Universitaria Senese (AOUS), Siena, Italy

**Keywords:** Abdomen, Laparotomy, Laparoscopy, Peritoneal neoplasms, Tomography (x-ray computed)

## Abstract

**Background:**

Computed tomography (CT) is widely used to diagnose peritoneal metastases (PM), with debated accuracy. Dual-energy CT (DECT) may improve accuracy, yet its diagnostic performance is still unknown. We explored the potential of DECT for PM detection and quantification.

**Materials and methods:**

We retrospectively included patients undergoing staging DECT for cancers with a high risk of peritoneal involvement, followed by staging laparoscopy/laparotomy, which served as the reference standard. Nine readers with varying experience levels (three expert, three intermediate, and three inexpert) reviewed two sets of images, separated by ≥ 60 days, considering the presence/absence of PM, abdominal region(s) involved, and calculated the radiological peritoneal cancer index (PCI). The first set included contrast-enhanced delayed-DECT scans reconstructed as virtual 120-kVp images; the second set also included virtual monoenergetic, 40-keV images and iodine maps. Performance metrics, receiver operating characteristic (ROC) analysis, McNemar, DeLong, and Wilcoxon tests were applied.

**Results:**

Twenty patients (mean age 64.2 years; 12 females) were included, 10 with PM. At per-patient analysis, the addition of monoenergetic 40-keV images and iodine maps slightly increased the performance and improved inter-reader agreement, with significant benefit for inexperienced readers only (*p* = 0.010). Per-region analysis demonstrated a significant advantage with an area under the ROC curve ranging from 0.709 to 0.766 (*p* < 0.001), confirmed for each reader group; in addition, the inter-reader agreement significantly improved. Quantitative analysis showed a reduction in the differences between CT results and surgical PCI by DECT (4 ± 12 *versus* 2 ± 9, *p* < 0.001).

**Conclusion:**

DECT-derived reconstructions in the delayed-phase enhanced PM detection and quantification.

**Relevance statement:**

Delayed-phase DECT reconstruction showed superior accuracy over conventional CT in detecting and quantifying peritoneal metastases. These findings could help establish a new standard CT protocol for malignancies with peritoneal tropism.

**Key Points:**

- CT is the most widely used technique for assessing peritoneal metastases.
- The accuracy of CT for peritoneal metastases is debated; dual-energy CT shows promise.
- In our study, delayed-phase dual-energy CT provided significant advantages for all readers.

**Graphical Abstract:**

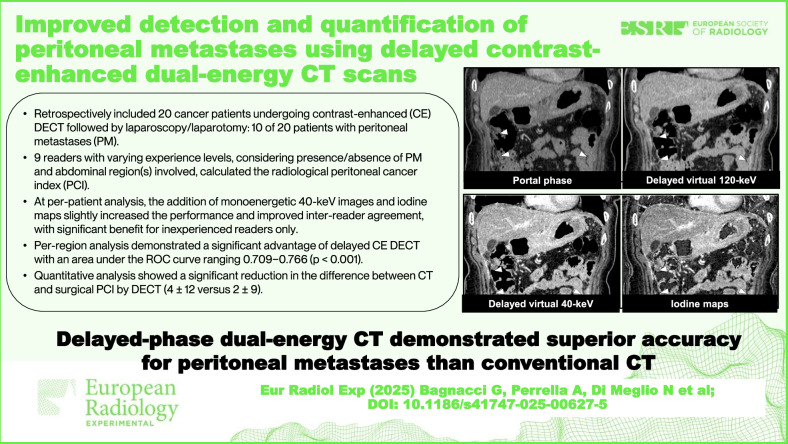

## Background

Peritoneal metastasis (PM), also known as peritoneal carcinosis or carcinomatosis [1], refers to the dissemination of neoplastic cells into the peritoneal cavity [2]. This process most commonly occurs in primary tumors such as ovarian, gastric, and colorectal cancers, but can also occur in other malignancies. The frequency of peritoneal involvement varies among different types of cancer. Regardless of the cancer type, its presence, whether synchronous or metachronous [3], remains consistently associated with poor prognosis [4–7]. In addition to the presence, the extent of PM represents a critical determinant that significantly influences patient survival and guides clinical management strategies. Approximately 30 years ago, Jacquet and Sugarbaker [8] introduced the peritoneal cancer index (PCI), a numerical score to grade the burden and describe the distribution of PM in thirteen abdominal regions. Initially developed for laparotomic assessment, the PCI was shown to correlate strongly with prognosis and was later adapted for laparoscopic assessment [9]. Over time, various imaging modalities, including computed tomography (CT), positron emission tomography, and magnetic resonance imaging (MRI), have been explored in the challenging quest to accurately predict PCI scores [10, 11].

Accurate quantification of PCI has gained critical importance in recent years, as multiple cutoff values have been proposed in the literature for different primary malignancies. These thresholds are used to assess resectability and operability, taking into account not only the technical feasibility of surgery but also the different prognostic implications associated with each neoplasm [12–14].

Furthermore, with the advent of new locoregional therapeutic strategies for PM, such as hyperthermic intraperitoneal chemotherapy (HIPEC) and pressurized intraperitoneal aerosol chemotherapy (PIPAC), imaging has gained an increasingly critical role. It is essential not only for the initial assessment of disease extent but also for monitoring therapeutic response and guiding subsequent management decisions.

Dual-energy CT (DECT) is an advanced imaging technique that has been shown to offer significant advantages in the assessment of a range of abdominal and oncological conditions [15]. In particular, DECT provides enhanced contrast resolution without compromising spatial resolution. One of the main challenges of CT in the detection of PM lies in the limited contrast resolution, relevant when attenuation differences between tissues are subtle, despite the excellent spatial resolution. Recent studies have begun to explore the potential of DECT in the assessment of peritoneal malignancies, highlighting its added value. One particularly compelling observation is the tendency of PM to exhibit delayed-phase contrast enhancement [16].

By exploiting this property, DECT has the potential to significantly enhance the conspicuity of PM on delayed-phase images, allowing radiologists to make more accurate and confident diagnoses. Accordingly, the objective of this study is to assess the effectiveness of delayed-phase DECT in improving the detection and quantification of PM, regardless of the level of experience of radiologists.

## Materials and methods

### Patient selection process

Institutional review board approval was obtained, and written informed consent was acquired for this retrospective study. CT scans of 98 patients, performed between September 2020 and July 2022, were collected from our radiology database. The inclusion criteria were: (1) primary or metastatic abdominal neoplasm mentioned in the radiological report; (2) availability of an adequate presurgical CT that included a dual-energy (DE) acquisition performed in the delayed phase (DP), acquired 180–300 s after venous injection of nonionic iodinated contrast. The following exclusion criteria were applied: (1) lack of surgical PM and/or PCI evaluation; (2) surgical evaluation performed more than 30 days after CT scan; (3) CT performed during or after neoadjuvant chemotherapy or locoregional peritoneal treatment.

Among patients without PM confirmation, a group of patients were selected to form a control group with the following selection criteria: (1) negative laparoscopy within 30 days of CT confirmed histologically and by negative peritoneal washing; (2) high risk of PM development based on the primary site and stage; (3) patients with previous abdominal surgery for benign conditions were preferred.

**Fig. 1:**
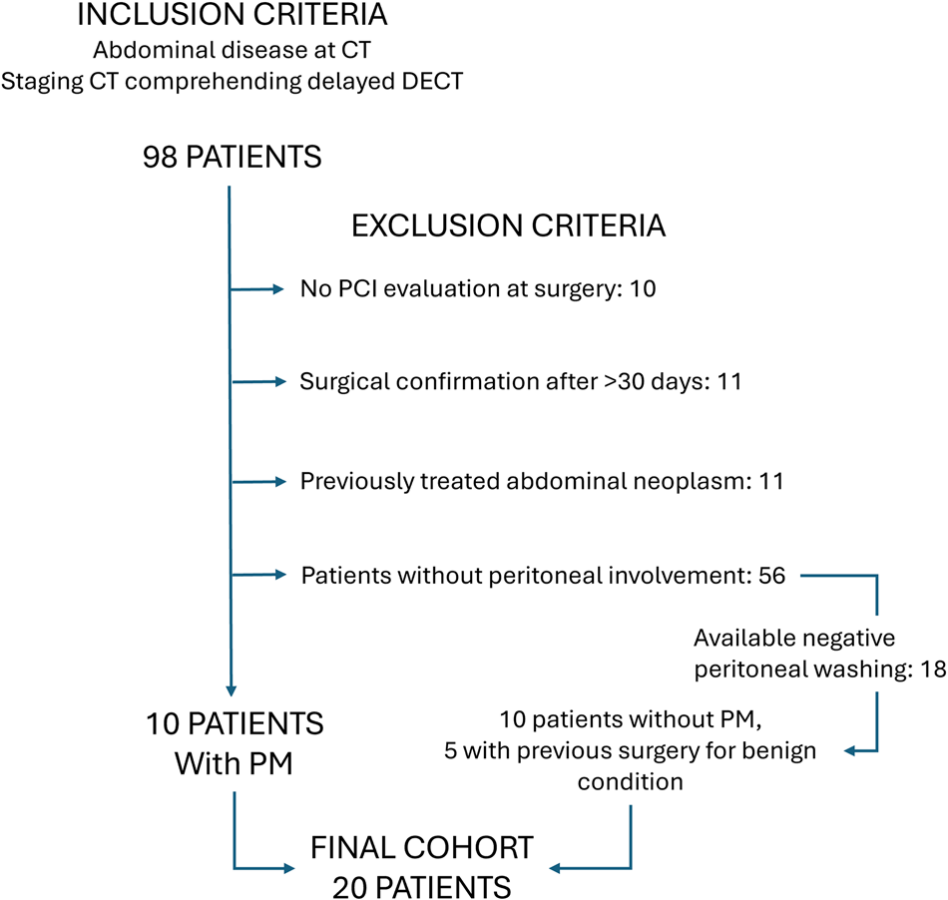
Flowchart illustrating the patient selection process. CT, Computed tomography; DECT, Dual-energy CT; PCI, Peritoneal cancer index; PM, Peritoneal metastases

The entire patient selection process is detailed in Fig. 1.

### CT protocol

All patients underwent a contrast-enhanced CT examination using a Discovery CT 750-HD-scanner (GE Healthcare) equipped with a rapid-switching dual-energy technology. Acquisition parameters are described in Table 1.

**Table 1:** Acquisition parameters

	Arterial phase	Portal phase	Delayed-phase dual-energy
Scan type	Helical	Helical	Helical
Slice thickness (mm)	1.25	2.5	2.5
Interval reconstruction (mm)	0.6	1.2	1.2
Tube voltage (kV)	120–140	120	80/140
Tube current (mA)	250–550*	250–550*	550
Rotation time (s)	0.6	0.5	0.6
Beam pitch (mm/s)	0.984/1	0.984/1	0.984/1
Detector coverage (mm)	40	40	40
Index noise (%)	16	16	16
Scan field of view (mm)	50	50	50
Reconstruction kernel	Soft	Soft	Soft
Acquisition time delay (s)	35–45	60–75	180–300

* The mA values were adjusted using the Automatic Exposure Control system

Contrast-enhanced images were acquired after the injection of nonionic iodinated contrast agent (3–3.5 mL/s, 1.5–1.8 mL/kg of Iopamidol/Iopamiro370), using a power injector. All studies included at least two helical acquisitions, one in the portal phase (PP), scanned 70–80 s after contrast injection, and the other one in the DP, scanned 180–300 s after contrast injection, the latter performed with dual-energy mode. In 12 of 20 examinations, a thin-slice (1.25-mm thickness) abdominal arterial phase (AP) scan was also performed. In 4 of 20 examinations, a hyperosmotic polyethylene glycol solution (SELGesse, approximately 1.5 L) was administered to achieve adequate distension of the bowel loops.

Following the acquisition of the DECT scans, postprocessing techniques were used to generate a 120-kVp-like image, a virtual monoenergetic image (VMI) with a keV value of 40 (VMI-40 keV), and a material density iodine map. Postprocessing was performed on a dedicated workstation (Advantage Workstation 4.6, GE Healthcare).

### Surgical exploration

Surgical exploration of the abdominal cavity was performed in five cases by exploratory laparotomy. In fifteen patients (five with PM), videolaparoscopy was performed by an expert surgeon (D.M.). Photographs and videos of surgical procedures were reviewed by a radiologist (G.B.) and a surgeon (S.P.) to ensure the accuracy and the agreement of the PCI calculated in the operating room.

### Study workflow

The images of the selected patients were anonymized and then uploaded to the Picture Archiving and Communication System. Two sets of images were uploaded for each patient: (1) one including unenhanced and contrast-enhanced AP, PP, and DP with only the 120-kVp simulated reconstruction; (2) another one including all the previous series with the addition of VMI-40-keV and iodine maps derived from the delayed-phase (DP) scan.

Both sets of images were read by 9 radiologists with 3 different levels of experience in abdominal imaging: (1) three inexperienced readers, *i.e*., radiology residents with 2 years of experience in abdominal imaging (C.I., S.R., V.D.M.); (2) three readers with intermediate experience, *i.e*., 4th-year of radiology residency (L.S., N.D.M., A.P.); (3) three senior expert radiologists with 9, 10, and 20 years of experience in abdominal imaging (F.G., S.G., M.A.M.)

Readers were also provided with didactic slides on peritoneal anatomy and a scheme for calculating the radiological PCI prior to image analysis. A special tutorial was prepared for readers with limited experience. The first set of images (“standard CT”) was provided to the readers. After a minimum interval of 60 days, the second set of delayed-phase 120-kVp virtual scans was supplemented with VMI-40-keV and iodine maps. All images were independently reviewed by nine radiologists, who were blinded to the surgical and histological results. The only information provided was that not all patients had peritoneal involvement, and the major abdominal surgical procedures they had undergone were listed.

During image analysis, a data collection form was used to record responses regarding the presence or absence of PM and the calculation of PCI.

**Fig. 2:**
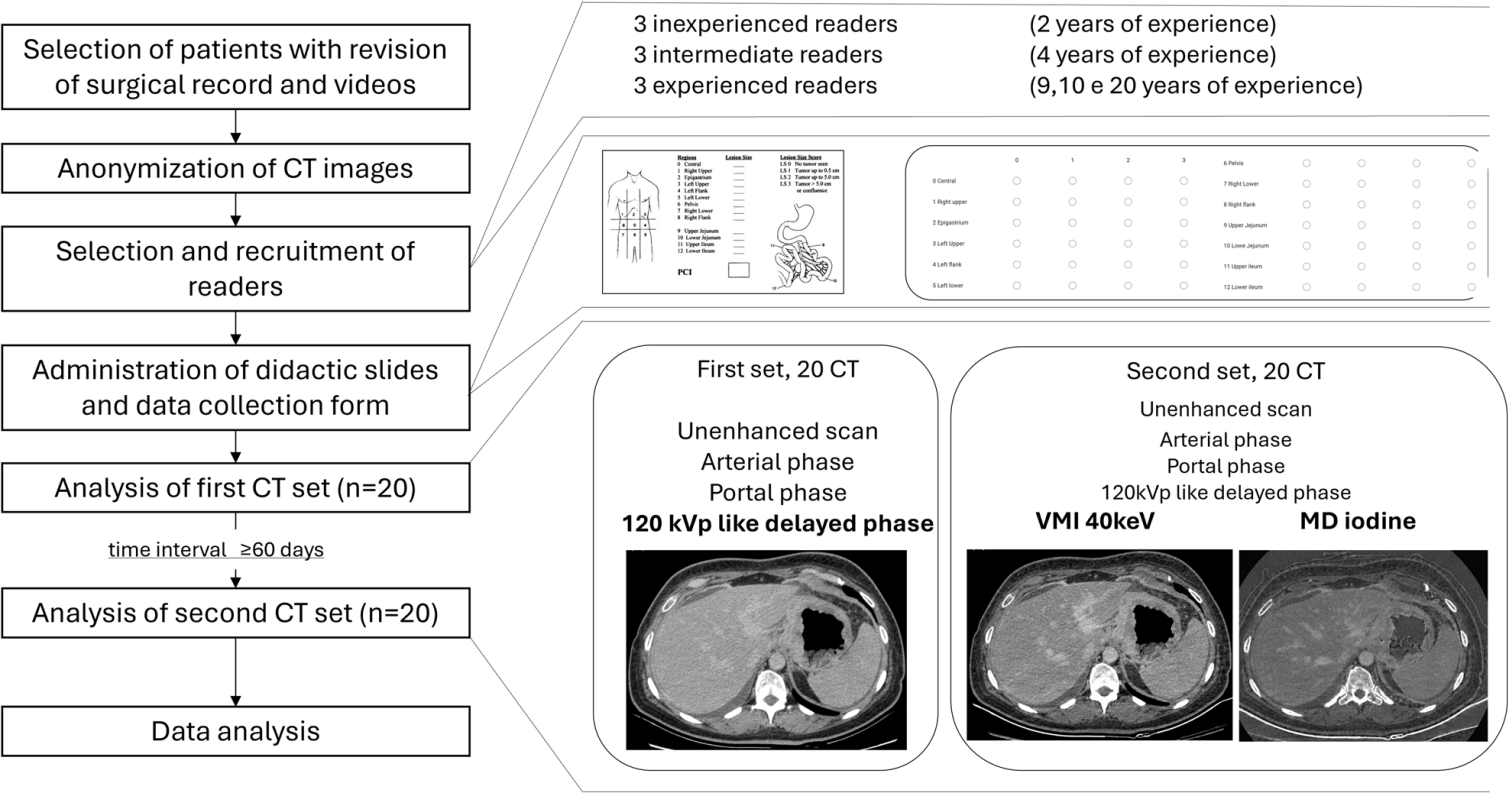
Study flowchart. CT, Computed tomography; MD, Material decomposition; VMI, Virtual monoenergetic images

The workflow is summarized in Fig. 2, and detailed guidelines for image analysis, including examples of PCI evaluation on CT, are available in the Supplementary materials (Figs. S2, S3).

### Statistical analysis

The accuracy in the identification of PM was assessed through contingency tables and the analysis of sensitivity, specificity, positive predictive value, negative predictive value, false-positive rate, false negative rate, positive likelihood ratio, negative likelihood ratio, diagnostic odds ratio (DOR). The agreement among readers was evaluated through the Fleiss κ [17].

The quantification of PM was assessed both as the presence/absence of PM in each PCI quadrant and as the numerical burden of disease according to the rules of quantification of the PCI.

The identification of PM in different patients and regions was analyzed using the same statistical methods, including the construction of contingency tables and the calculation of performance metrics. The McNemar and DeLong tests were used to identify significant differences and to compare reader performance. Means, medians, interquartile range and standard deviations regarding the numeric PCI differences were used for quantification. The distribution was assessed using the Shapiro-Wilk test, and the Wilcoxon paired test was applied to compare the medians. Boxplot and Bland–Altman plot were built up.

SPSS v.26 and RStudio v.4.2.1 were employed for statistical analysis.

## Results

### Population

**Fig. 3:**
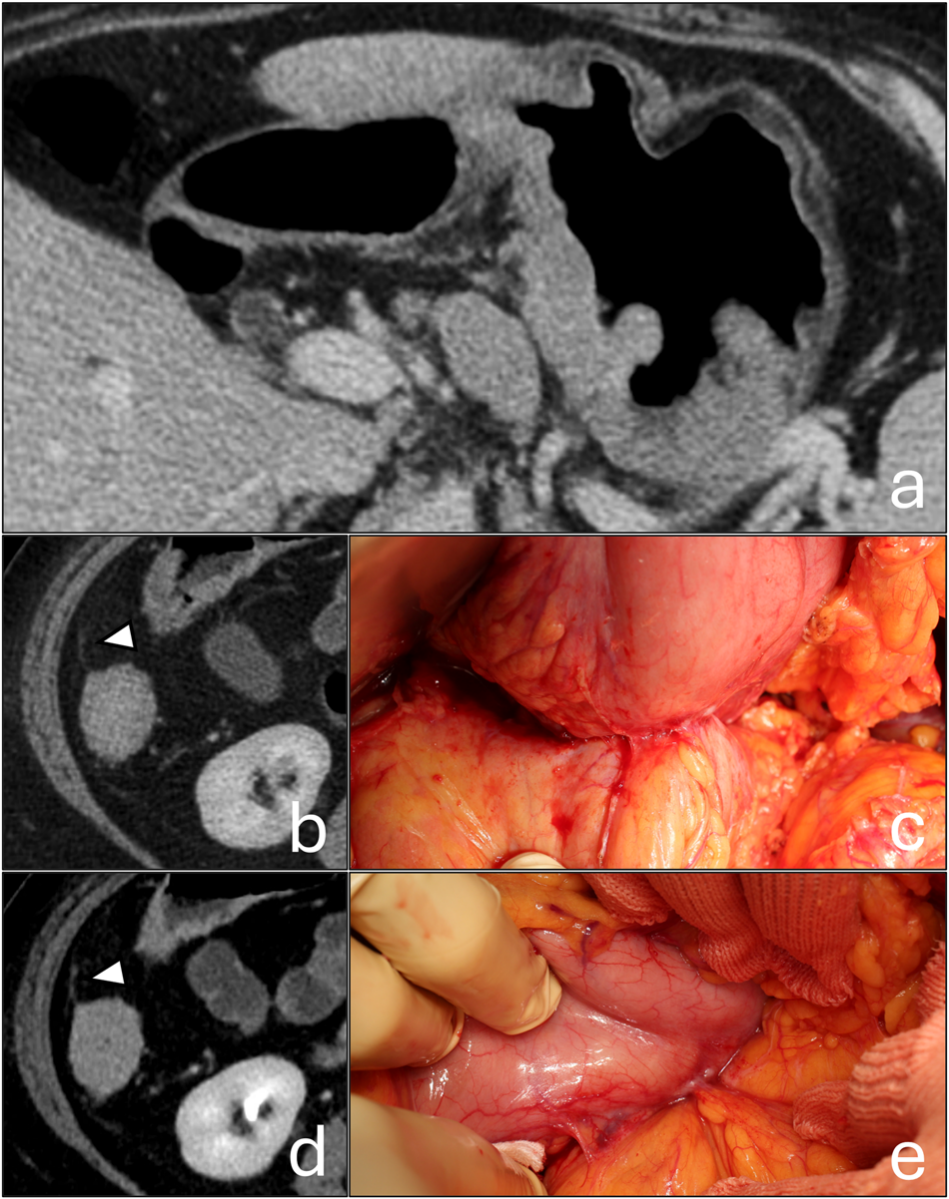
The patient exhibited the highest false-positive rate among the readers. **a** Wide gastric neoplasm with large lymph nodes and extraparietal infiltration. Virtual 120 kVp (**b**) and VMI-40-keV (**d**) show a thickening of the peritoneal lining next to the ascending colon (arrowheads). Note that no significant enhancement can be appreciated in the delayed phase, not even with VMI-40-keV. **c** and **e** Intraoperative evidence of extensive adhesion bands. The patient had undergone an appendectomy and cholecystectomy 40 years ago, and the history indicated that the procedure was “complex”

According to the selection criteria, 20 patients were included, 10 with PM and 10 without. A total of 260 peritoneal regions were assessed. The mean age of the patients was 64.2 ± 16.9 years (mean ± standard deviation), and 12 of them were female. The characteristics of the patients and those of the primary neoplasms are summarized in Table 2. Among the patients without PM, five had undergone previous abdominal surgeries: cholecystectomy in three cases, previous surgery for mechanical obstruction in one case, cholecystectomy and appendicectomy in one case (Fig. 3). The median time between staging CT and the surgical procedure was 10 days (IQR 4–17), with a range of 1 to 30 days.

**Table 2:** Demographics and clinical characteristics of patients enrolled

Age (years)	Sex	Primary tumor location	Grade	Clinical stage*	Histological data	Surgical PCI
88	M	Stomach	G3	cT4bN3bMx	Tubular Adk	0
55	M	Stomach	G2	cT4aN+M+	Tubular and poorly cohesive Adk	0
82	M	Stomach	G2	cT4aN3Mx	Tubular Adk	0
49	F	Stomach	G2	cT3/T4aN+Mx	Adk NOS	0
61	M	Stomach	G3	cT4aN3Mx	Tubular Adk	0
75	F	Stomach	G3	cT4bN3M+	Poorly cohesive Adk	0
26	F	Stomach	G3	cT4aN+Mx	Tubular Adk	0
53	F	Stomach	G3	cT4aN+Mx	Poorly cohesive Adk	0
58	F	Colon	G2	cT4N + M0	Adk NOS	0
58	M	Stomach	G3	cT4aN+Mx	Poorly cohesive Adk	0
53	F	Pancreas	G2	cT4N+M+	Ductal Adk	18
63	M	Stomach	G3	cT4aN+M+	Poorly cohesive and SRC Adk	22
56	F	Endometrium	G3	(T excised) Mx	Endometrioid arcinoma	4
60	F	Ovary	G2	cT3N0M+	Serous carcinoma	30
87	F	Breast	/	(T excised) M+	Lobular arcinoma	24
46	F	Ovary	G3	cT3N0M+	Mucinous Adk	30
61	F	Stomach	G3	cT4aN+M+	Tubular Adk	25
93	F	Stomach	/	cT4aN+Mx	Tubular Adk	2
63	M	Stomach	G2	cT4aN+M+	Adk NOS	26
89	F	Stomach	G3	cT4aN+M+	Poorly cohesive and SRC Adk	25

*Adk* Adenocarcinoma, *M+* Peritoneal metastases probable at the original radiological report, *Mx* Peritoneal metastases possible at the original radiological report, *NOS* Not otherwise specified, *PCI* Peritoneal cancer index, *SRC* Signet ring cells

* The clinical stage refers to the original radiological report

### Per-patient analysis

The analysis focused on determining the presence or absence of PM in individual patients. Table 3 provides a detailed comparison of reader performance using DECT *versus* standard CT. No significant differences between the diagnostic performance of DECT and standard CT were found (*p* = 0.607). Similarly, no significant differences were observed for DECT *versus* standard CT when the analysis was stratified according to the level of experience of the readers: inexpert (*p* = 0.610), intermediate (*p* = 0.579), and expert (*p* = 0.683).

**Table 3:** Results of the patient-based assessment of peritoneal metastases

	Sensitivity	Specificity	PPV	NPV	Accuracy	LR+	LR-	DOR
Standard CT	81%	73%	75%	79%	77%	2.98	0.26	7.5
DECT	83%	79%	80%	83%	81%	3.95	0.21	18.7
Inexpert, CT	78%	57%	64%	72%	68%	1.81	0.38	4.7
Inexpert, DECT	80%	70%	73%	78%	75%	2.67	0.29	9.3
Intermediate, CT	78%	70%	72%	76%	74%	2.61	0.31	5.5
Intermediate, DECT	83%	73%	76%	81%	78%	3.13	0.23	13.8
Expert, CT	87%	92%	91%	87%	89%	10.4	0.15	71.5
Expert, DECT	87%	93%	93%	88%	90%	13	0.14	91

*CT* Computed tomography, *DECT* Dual-energy CT, *DOR* Diagnostic odds ratio, *LR+* Likelihood ratio positive, *LR-* Likelihood ratio negative, *NPV* Negative predictive value, *PPV* Positive predictive value

A significantly higher area under the curve for DECT compared to standard CT (0.811 *versus* 0.728, *p* = 0.002) was found. When stratified by reader’s experience, a significant improvement was observed for inexperienced readers, with the AUC increasing from 0.60 to 0.75 (*p* = 0.010). However, no significant differences were observed for intermediate (*p* = 0.055) or expert readers (*p* = 0.568).

Regarding inter-reader agreement, inexperienced readers showed an improvement from 0.727 (95% CI: 0.723–0.732) to 0.786 (95% CI: 0.782–0.790), intermediate readers from 0.840 (95% CI: 0.836–0.844) to 0.866 (95% CI: 0.862–0.871) and experienced readers from 0.920 (95% CI: 0.916–0.924) to 0.947 (95% CI: 0.942–0.951).

### Per-region analysis

**Fig. 4:**
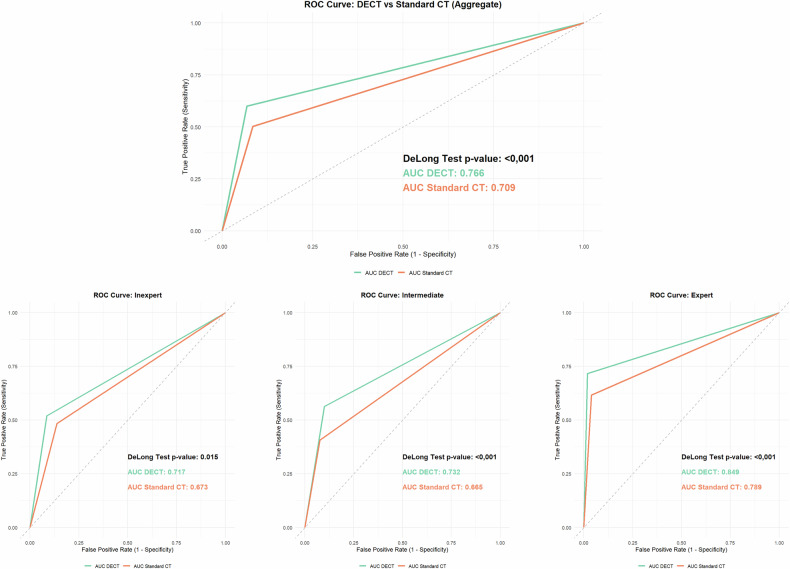
Receiver operating characteristics (ROC) analysis for the region-based assessment. Note the significant benefit for readers of all experience levels

Per-region analysis assessed the presence or absence of PM in each region as defined by Jacquet and Sugarbaker, with performance measures summarized in Table 4. McNemar test showed significant differences between DECT and standard CT, with overall *p*-values of < 0.001 and stratified results showing significance for all levels of experience (inexperienced *p* = 0.036, intermediate *p* < 0.001, expert *p* = 0.029). An improvement in the area under the curve across all levels of experience of the readers was observed (Fig. 4).

**Table 4:** Results of the region-based assessment of peritoneal metastases

	Sensitivity	Specificity	PPV	NPV	Accuracy	LR+	LR-	DOR
Standard CT	50%	92%	79%	75%	76%	5.9	0.5	10.9
DECT	60%	93%	85%	79%	80%	8.8	0.4	20.5
Inexpert, CT	48%	86%	69%	73%	72%	3.5	0.6	5.9
Inexpert, DECT	52%	91%	79%	75%	76%	6.1	0.5	11.6
Intermediate, CT	41%	92%	77%	71%	72%	5.3	0.6	8.2
Intermediate, DECT	56%	90%	78%	77%	77%	5.6	0.5	11.6
Expert, CT	62%	96%	91%	80%	83%	15.6	0.4	39
Expert, DECT	72%	98%	96%	85%	88%	38.2	0.3	132.4

*CT* Computed tomography, *DECT* Dual-energy CT, *DOR* Diagnostic odds ratio, *LR+* Likelihood ratio positive, *LR-* Likelihood ratio negative, *NPV* Negative predictive value, *PPV* Positive predictive value

Inter-reader agreement also improved with DECT. Inexperienced readers showed an increase in agreement from 0.286 (95% CI: 0.284–0.288) to 0.440 (95% CI: 0.438–0.442), intermediate readers from 0.542 (95% CI: 0.540–0.545) to 0.633 (95% CI: 0.631–0.635), and expert readers from 0.618 (95% CI: 0.616–0.620) to 0.626 (95% CI: 0.623–0.628).

Excluding regions 9–12, which correspond to bowel loops, accuracy improved from 78% to 80% overall, from 74% to 75% for inexperienced readers, from 74% to 79% for intermediate readers, and from 86% to 91% for experienced readers. Specific analysis for regions 0–8 and 9–12 is reported in Tables S1 and S2.

### Quantitative PCI analysis

**Fig. 5:**
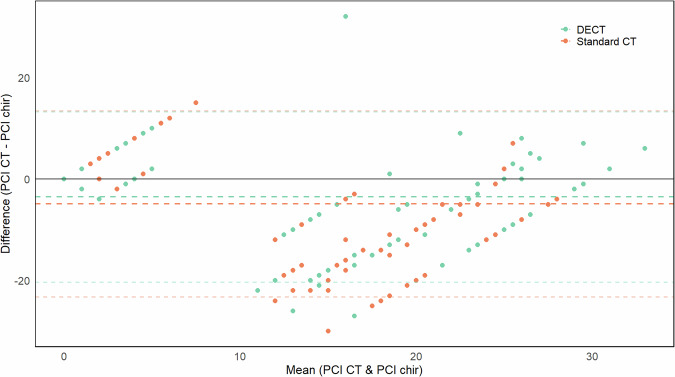
Bland–Altman plot: the smaller difference between DECT and PCI is evident from the DECT line (green, dashed), which is closer to 0. Additionally, the distance between the more nuanced dashed lines is smaller for DECT, indicating less variance across readers. CT, Computed tomography; DECT, Dual-energy CT; PCI, Peritoneal cancer index

**Fig. 6:**
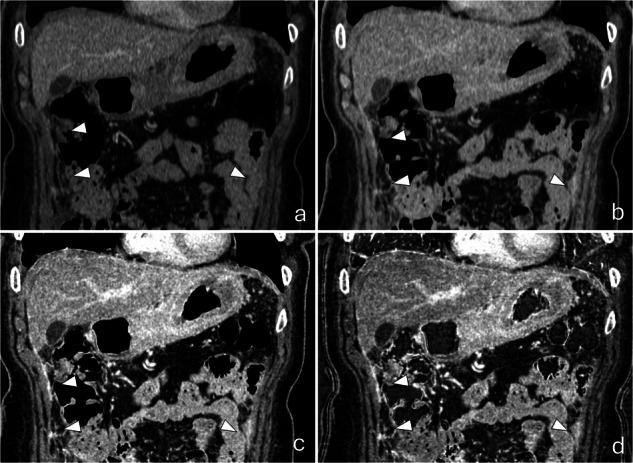
Example of DECT reconstruction advantages. Portal phase (**a**), delayed virtual 120-kV (**b**), delayed VMI-40-keV (**c**), iodine maps (**d**). Patients showed PM in regions 1, 8, and 4 (arrowheads). There were differences in readers’ calculation of PCI when employing DECT: the mean PCI was 1.3 *versus* 2.9 for region 1, 0.6 *versus* 1.8 for region 8, and 1.0 *versus* 1.7. for region 4. CT, Computed tomography; DECT, Dual-energy CT; PCI, Peritoneal cancer index; PM, Peritoneal metastases

The median difference between surgical PCI and CT PCI was 4 (IQR 0–12) for standard CT and 2 (IQR 0–9) for DECT (*p* < 0.001). When stratified by experience level, the median differences between surgical and CT PCI improved significantly: inexperienced readers showed a reduction from 5 (IQR 0–10) to 4 (IQR 0–10) (*p* = 0.040), intermediate readers from 2.5 (IQR 0–14.5) to 2 (IQR 0–9) (*p* = 0.060), and expert readers from 2 (IQR 0–10) to 0.5 (IQR 0–6) (*p* = 0.002). Bland–Altman plot, illustrating the differences between DECT and standard CT, is presented in Fig. 5. In Fig. 6, a practical example of the advantages is provided.

Original radar graphics for the eight patients with the highest burden of disease are shown in Supplementary Fig. S1. A subanalysis focusing on regions with PCI = 1 showed an improvement in sensitivity across all levels of experience with DECT, without compromising specificity. Sensitivity increased from 10% to 24% for inexperienced readers, from 38% to 48% for intermediate readers, and from 39% to 58% for expert readers.

## Discussion

This study supports the combination of DECT DP scan and DECT reconstructions as a promising protocol for the evaluation of PM. The study was conducted using a per-patient, per-region (according to the scheme by Jacquet and Sugarbaker), and quantitative analysis approach.

Per-patient analysis showed a significant advantage when all readers were considered together using the DeLong test. However, the McNemar test did not show a significant difference, suggesting that the improvement, although present, may be limited. After stratification by experience level, a significant benefit was observed only for inexpert readers, likely due to the limited size of the cohort and the consequent risk of a type II error due to lack of statistical power. However, a moderate improvement in diagnostic metrics and inter-reader agreement was also observed among readers with intermediate and high levels of experience. In particular, DECT slightly increased specificity for expert readers and improved both sensitivity and specificity for intermediate readers.

Per-region analysis showed a significant improvement in performance for all readers. This finding contrasts with the results by Rodolfino et al [18], who showed no significant benefit of delayed scanning for experienced radiologists. This discrepancy is likely attributable to the use of DE reconstructions in our study, which increased sensitivity, particularly in low disease burden regions (PCI = 1), where expert sensitivity increased from 39% to 58%. Moreover, unlike Rodolfino et al, who included only ovarian cancer cases, our study also encompassed neoplasms originating from other primary sites, which may have influenced the degree of delayed enhancement of PM.

Quantitative analysis revealed a tendency among readers to underestimate disease burden. However, this estimation improved significantly with DECT, reaching a relatively high level of accuracy for expert readers with a difference of 0.5 ± 6 (mean ± standard deviation). Nevertheless, some challenges remain, including a high variance in PCI quantification and good, albeit suboptimal, intra-reader agreement. While these issues may be partly due to inherent limitations of the imaging modality and reader expertise, other contributing factors are plausible. In particular, the majority of patients did not undergo small bowel distension, as the examinations were primarily performed for staging purposes and only occasionally PM was strongly suspected (4 of 20). In addition, suboptimal slice thickness (2.5 mm instead of 1.25 mm) and limited familiarity with DECT imaging, even among expert readers, may have further impacted their performance.

Compared to the studies of conventional CT, our DECT protocol appears to improve the diagnostic performance of radiologists, even when considering only studies with rigorous methodologies, including contrast-enhanced imaging, slice thickness ≤ 3 mm, and grading/assessment by expert readers [19–21].

These findings may be attributed to the characteristics of both PM and DECT. On the one side, PM exhibits DP enhancement, as detailed in previous studies, including that by Chia et al in 2022 [16]. This phenomenon may be explained by the distinctive interaction between cancer cells and peritoneum, often resulting in a pronounced infiltration of cancer-associated fibroblasts [22]. Such biological peculiarities are likely to contribute to the delayed enhancement patterns that make PM more detectable with our protocol. On the other hand, the well-established high iodine sensitivity of DECT provides a critical advantage in a low-contrast resolution setting, such as the peritoneal assessment. This sensitivity allows for better differentiation and visualization of subtle disease, particularly in regions of low disease burden or minimal enhancement.

Together, these factors may contribute to the improved diagnostic accuracy observed in our study. This is not the first report on the use of DECT for PM assessment. The potential of DECT for PM evaluation was first noted in a 2017 white paper by the Society of Computed Body Tomography and Magnetic Resonance [23]. The first available clinical study was published in 2019 by Darras et al [24], who demonstrated that 40-keV DECT reconstructions significantly improved lesion conspicuity and diagnostic confidence. These findings are consistent with our observations and further reinforce the value of DECT revealing PM.

In 2020, Lennartz et al [25] investigated the role of iodine overlays and reported improved specificity when these were evaluated alongside conventional images. Although our study used different reconstructions, we also observed a slight improvement in specificity. More recently, Pisuchpen et al [26] highlighted the potential utility of iodine maps, demonstrating their advantages for readers of all levels of experience in detecting PM. Our results are consistent with these findings; however, our readers showed a preference for 40-keV reconstructions over iodine maps. This preference may be due to limited familiarity with iodine maps. In addition, the lower keV level selected for our study (40 keV *versus* 65 keV by Pisuchpen study) likely provided sufficient iodine enhancement to make iodine maps less meaningful.

Compared to previous studies on DECT, we incorporated a per-region and quantitative analysis of the PCI, which adds significant value given the prognostic and management implications of disease burden estimation. Including this information in radiological evaluations aligns with recent recommendations on imaging of PM from European and international societies [27].

Furthermore, all previous studies utilized the portal or a similar acquisition phase, whereas our study identified the delayed phase as the optimal imaging phase. The statistical methodology employed in our study not only strengthens the validity of our findings but also provides a framework that can be utilized for future meta-analyses and comparisons between different imaging modalities.

Indeed, PM can also be evaluated using alternative imaging modalities, particularly positron emission tomography-CT and MRI. To date, two meta-analyses have compared the diagnostic performance of these techniques for the PM assessment. In 2017, Laghi et al [10] concluded that CT remains the most robust and reliable technique. Conversely, in 2020, Van Saint et al [11] identified MRI as the preferred modality for PM assessment. Although our study did not include a direct comparison with MRI, the diagnostic performance of DECT in our cohort, particularly when interpreted by experienced readers, resulted in a diagnostic odds ratio (DOR) of 132. While caution is warranted when comparing data from different studies, this value appears higher than the DOR of 63 reported for MRI in the meta-analysis by Van Saint et al [11], which may suggest that DECT could offer comparable or potentially superior diagnostic performance in selected conditions.

The present study has several limitations. The patient cohort is relatively small; however, the selection process was carried out meticulously, the analysis was also performed on a regional basis, the number of readers is high, and the presence of PM was histologically confirmed in all cases. In addition, CT scans were performed within 30 days of surgery, ensuring a close temporal relationship between imaging and surgical findings. In patients who were negative for PM on laparoscopy, the absence of disease was further validated by peritoneal washing cytology, enhancing the reliability of the negative findings. The inclusion of patients with a history of abdominal surgery reflects a “real-life” clinical scenario, where multiple confounding factors are present. While this adds complexity to the evaluation of negative cases, our findings demonstrate that DECT reconstruction does not lead to an increase in false positives.

The majority of case series included patients with gastric cancer, as our institution is a high-volume reference center for the management of this disease. Therefore, further validation of the results is needed to extend their validity to other primary neoplasms.

Furthermore, histological confirmation of the presence of PM in all sites identified during surgical exploration was not feasible. Nonetheless, it is reasonable to assume that neoplastic cells were present at the identified sites, as none of the patients were undergoing chemotherapy or immunotherapy at the time of the CT scan and of surgical exploration, minimizing the likelihood of false-positive findings due to treatment effects. It is plausible that readers with limited experience improved their performance during the visual interpretation of the images. The improved accuracy observed with the VMI-40-keV and iodine maps reconstructed images may, in part, be attributed to their growing familiarity with imaging features. The fixed order of image interpretation (first and second set) may have contributed to a slight improvement in performance for the second set due to experience bias. However, the long interval between the two reading sessions likely mitigated this effect. It is also possible that different orders of presentation could have introduced other types of bias.

The wide range of delay times (180–300 s) is related to the retrospective nature of the study and the absence of a precisely defined protocol at the time of image acquisition. Based on our findings, a delay of approximately 200–240 s may represent an optimal window for future studies, as it allows sufficient contrast uptake in fibrous tissue (such as that associated with PM), while minimizing bladder filling, which could impair pelvic evaluation.

Finally, only 4 out of 20 patients underwent bowel loop distention, and only 12 out of 20 had AP images with a 1.25 mm thickness. Although both bowel loop distention and thin-slice acquisition are potentially significant advantages, it should be emphasized that the DECT reconstructions still provided a clear benefit. The ability to compare the same examination from the same patient significantly reduces potential bias arising from small inter-patient methodological differences.

Despite the limitations, the overall results are promising. The use of VMI-40-keV reconstructions and iodine maps from DECT in the DP significantly improved the detection and quantification of PM. If validated in larger cohorts, these findings have the potential to establish a new standard CT protocol for the evaluation of malignancies with peritoneal tropism. Moreover, the improved lesion conspicuity achieved with this approach could not only facilitate radiological interpretation but also enhance future segmentation workflows, particularly when integrated with deep learning based tools [28]. Finally, with the advent of next-generation DECT and photon-counting CT scanners, which maximize spatial and contrast resolution, this protocol may re-establish CT as one of the most accurate imaging modalities for the assessment of PM.

## Supplementary information

**Additional file 1**: **Fig. S1** Original method developed for quantitative and qualitative representation of PCI scores. The graphs provide an intuitive visualization of the disease for the 8 patients with the highest burden. The central value represents PCI = 0, and the outermost circle represents PCI = 3. Peripheral numbers are the PCI regions. The surgical PCI is shown in bold blue, and the mean PCI of all readers obtained with standard CT and with DECT are compared (green and light blue). In orange, the PCI is calculated by the most experienced radiologist using DECT. Note how the average DECT area is consistently larger than the standard CT area and closer to the bold blue area. *CT* Computed tomography, *DECT* Dual-energy CT, *PCI* Peritoneal cancer index. **Fig. S2** Examples of peritoneal metastases (PM) measurement. **a** A plaque-like PM with the corresponding measurement (**b**). **c** A nodular PM with the corresponding measurement (**d**). Once the extent of the disease is defined, accurate measurement is straightforward. All images are virtual monochromatic images at 40 keV. **Fig. S3** Pseudonodular peritoneal thickening initially measured as an isolated finding (**a**, **b**). Oblique multiplanar reconstruction (**c**, **d**) shows confluence with a second nodule, increasing the lesion size to 50 mm and upgrading the regional PCI score from 2 to 3. **Table S1:** Results of the region-based assessment of peritoneal metastases for regions 0–8. **Table S2:** Results of the region-based assessment of peritoneal metastases for regions 9–12.

## Data Availability

The datasets used and/or analyzed during the current study are available from the corresponding author on reasonable request.

## Abbreviations

AP: Arterial phase
CI: Confidence interval
DECT: Dual-energy computed tomography
DOR: Diagnostic odds ratio
DP: Delayed phase
PCI: Peritoneal cancer index
PM: Peritoneal metastases
PP: Portal phase
VMI: Virtual monoenergetic images
