# Supplementary material for: Improved detection and quantification of peritoneal metastases using delayed contrast-enhanced dual-energy CT scans
Source: Eur Radiol Exp. 2025 Oct 8;9:101. doi: 10.1186/s41747-025-00627-5 (PMC12508340; doi:10.1186/s41747-025-00627-5)
Supplement: Supplementary file 1 — Additional file 1: Fig. S1 Original method developed for quantitative and qualitative representation of PCI scores. The graphs provide an intuitive visualization of the disease for the 8 patients with the highest burden. The central value represents PCI = 0, and the outermost circle represents PCI = 3. Peripheral numbers are the PCI regions. The surgical PCI is shown in bold blue, and the mean PCI of all readers obtained with standard CT and with DECT are compared (green and light blue). In orange, the PCI is calculated by the most experienced radiologist using DECT. Note how the average DECT area is consistently larger than the standard CT area and closer to the bold blue area. CT Computed tomography, DECT Dual-energy CT, PCI Peritoneal cancer index. Fig. S2 Examples of peritoneal metastases (PM) measurement. a A plaque-like PM with the corresponding measurement (b). c A nodular PM with the corresponding measurement (d). Once the extent of the disease is defined, accurate measurement is straightforward. All images are virtual monochromatic images at 40 keV. Fig. S3 Pseudonodular peritoneal thickening initially measured as an isolated finding (a, b). Oblique multiplanar reconstruction (c, d) shows confluence with a second nodule, increasing the lesion size to 50 mm and upgrading the regional PCI score from 2 to 3. Table S1: Results of the region-based assessment of peritoneal metastases for regions 0–8. Table S2: Results of the region-based assessment of peritoneal metastases for regions 9–12. [file 41747_2025_627_MOESM1_ESM.docx]

**Supplemental material**


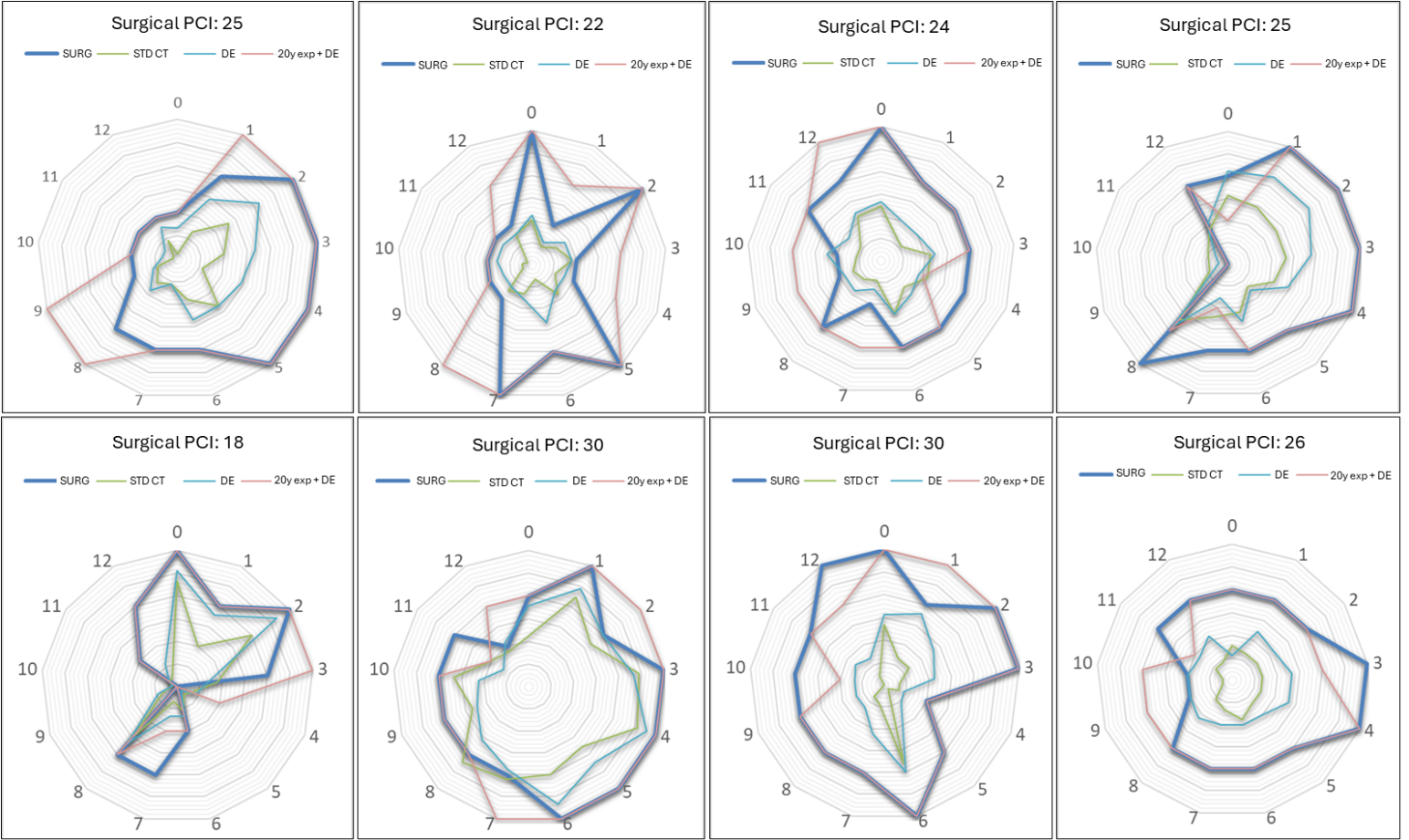


**Fig. S1** Original method developed for quantitative and qualitative representation of PCI scores. The graphs provide an intuitive visualization of the disease for the 8 patients with the highest burden. The central value represents PCI = 0 and the outermost circle represents PCI = 3. Peripheral numbers are the PCI regions. The surgical PCI is shown in bold blue, and the mean PCI of all readers obtained with standard CT and with DECT are compared (green and light blue). In orange, the PCI is calculated by the most experienced radiologist using DECT. Note how the average DECT area is consistently larger than the standard CT area and closer to the bold blue area. *CT* Computed tomography, *DECT* Dual-energy CT, *PCI* Peritoneal cancer index,

**Table S1:** Results of the region-based assessment of peritoneal metastases for regions 0-8

|  | Sensitivity | Specificity | PPV | NPV | Accuracy | LR+ | LR- | DOR |
| --- | --- | --- | --- | --- | --- | --- | --- | --- |
| Standard CT | 57% | 92% | 83% | 76% | 78% | 7.37 | 1.10 | 15.94 |
| DECT | 66% | 92% | 86% | 80% | 82% | 8.71 | 1.07 | 23.73 |
| Inexpert, CT | 49% | 91% | 78% | 72% | 74% | 5.28 | 1.08 | 9.44 |
| Inexpert, DECT | 51% | 91% | 80% | 73% | 75% | 5.86 | 1.09 | 10.95 |
| Intermediate, CT | 51% | 90% | 77% | 73% | 74% | 4.93 | 1.06 | 8.97 |
| Intermediate, DECT | 66% | 88% | 80% | 79% | 79% | 5.70 | 1.01 | 14.74 |
| Expert, CT | 72% | 96% | 93% | 84% | 86% | 19.30 | 1.11 | 66.70 |
| Expert, DECT | 81% | 98% | 96% | 88% | 91% | 32.61 | 1.08 | 169.86 |

*CT* Computed tomography, *DECT* Dual-energy CT, *DOR* Diagnostic odds ratio, *LR+* Likelihood ratio positive, *LR-* Likelihood ratio negative, *NPV* Negative predictive value, PPV Positive predictive value.

**Table S2:** Results of the region-based assessment of peritoneal metastases for regions 9-12

|  | Sensitivity | Specificity | PPV | NPV | Accuracy | LR+ | LR- | DOR |
| --- | --- | --- | --- | --- | --- | --- | --- | --- |
| Standard CT | 38% | 91% | 70% | 73% | 72% | 4.25 | 0.95 | 6.22 |
| DECT | 47% | 94% | 81% | 77% | 78% | 8.14 | 1.06 | 14.55 |
| Inexpert, CT | 49% | 81% | 58% | 75% | 70% | 2.56 | 0.78 | 4.03 |
| Inexpert, DECT | 52% | 92% | 77% | 78% | 78% | 6.38 | 0.99 | 12.31 |
| Intermediate, CT | 23% | 97% | 80% | 70% | 71% | 7.43 | 1.14 | 9.33 |
| Intermediate, DECT | 40% | 92% | 72% | 74% | 74% | 4.88 | 0.98 | 7.46 |
| Expert, CT | 42% | 95% | 83% | 75% | 77% | 9.08 | 1.10 | 14.91 |
| Expert, DECT | 50% | 99% | 96% | 78% | 82% | 48.29 | 1.23 | 94.68 |

*CT* Computed tomography, *DECT* Dual-energy CT, *DOR* Diagnostic odds ratio, *LR+* Likelihood ratio positive, *LR-* Likelihood ratio negative, *NPV* Negative predictive value, PPV Positive predictive value.

**PCI evaluation on CT**

Peritoneal metastases were assessed on CT scans according to the peritoneal cancer index (PCI) scheme proposed by Sugarbaker [1].

The distribution and implant size of the disease across 13 abdominopelvic regions were evaluated.For each region, the largest implant was scored on a scale from 0 to 3 as follows: i) 0 = no lesion identified; ii)1 = lesion up to 0.5 cm in maximum diameter; iii) 2 = lesion exceeding 0.5 cm but not exceeding 5 cm; iv) 3 = lesion or confluent lesions exceeding 5 cm. Confluent implants were considered a single lesion. The sum of the scores from all regions resulted in the total PCI score, ranging from 0 to 39. The same criteria were applied for both surgical and CT-based evaluations. Two-dimensional multiplanar reconstructions (coronal, sagittal, and oblique planes) were used to optimize the assessment of peritoneal recesses on CT imaging.

Peritoneal metastases may present with different morphologies. In most cases, they appear as nodules, plaques, or masses [2], which can be measured directly (Figs. S2 and S3). However, in specific sites such as the greater omentum and the small bowel mesentery, peritoneal metastases may manifest primarily as increased fat attenuation or a reticulonodular pattern, making direct measurement difficult. In these situations, readers were asked to determine whether the morphological changes were consistent with peritoneal metastases and, if so, to assign a PCI score based on the estimated extent of involvement.


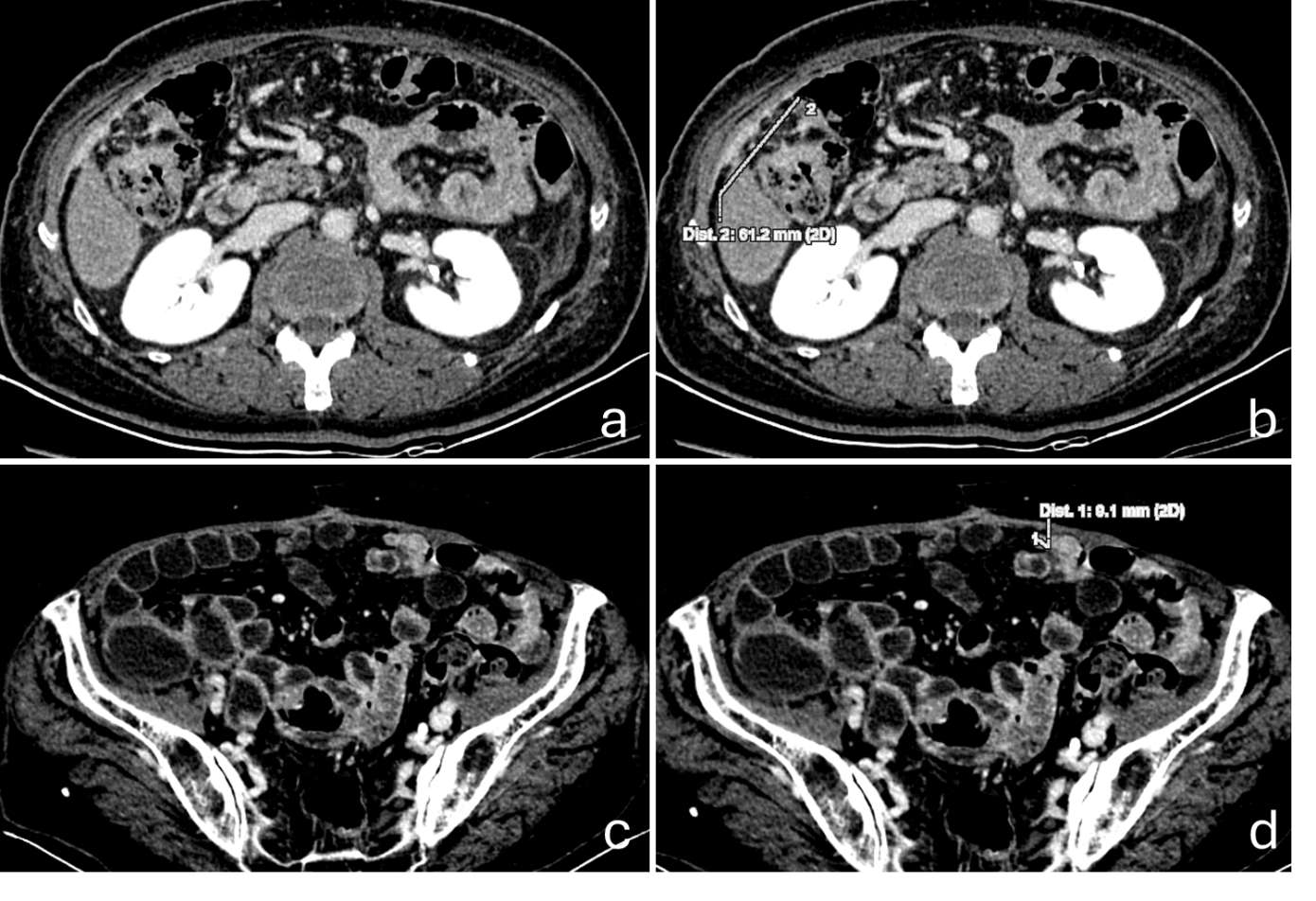


**Fig. S2** Examples of peritoneal metastases (PM) measurement. (**a**) A plaque-like PM with the corresponding measurement (**b**). (**c**) A nodular PM with the corresponding measurement (**d**). Once the extent of the disease is defined, accurate measurement is straightforward. All images are virtual monochromatic images at 40 keV.


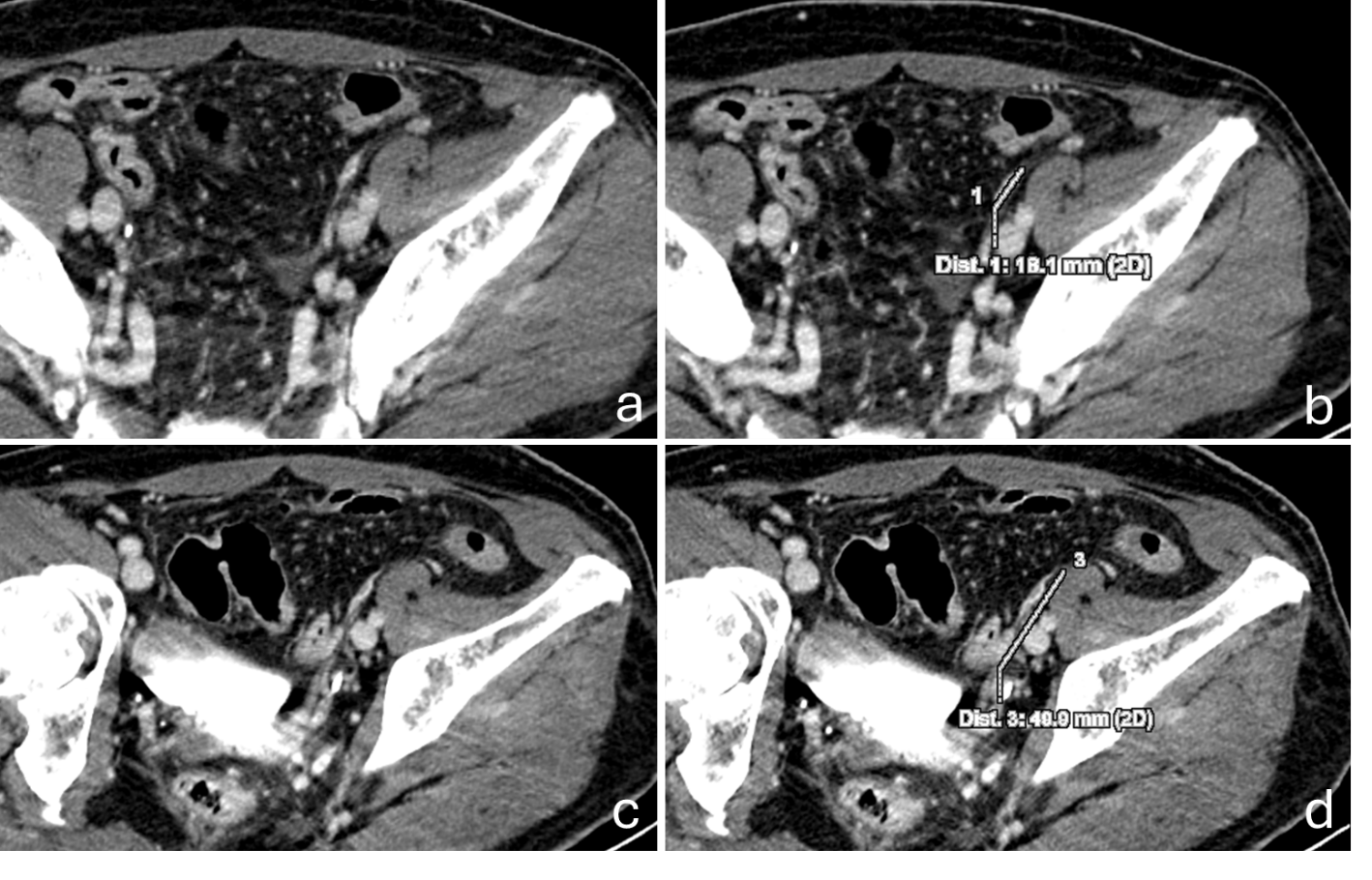


**Fig. S3** Pseudonodular peritoneal thickening initially measured as an isolated finding (**a**, **b**). Oblique multiplanar reconstruction (**c**, **d**) shows confluence with a second nodule, increasing the lesion size to 50 mm and upgrading the regional PCI score from 2 to 3.

**References**

- 1. Sugarbaker PH (2019) Laparoscopy in the diagnosis and treatment of peritoneal metastases. Ann Laparosc Endosc Surg. doi: 10.21037/ales.2019.04.04 [Ref 9 of main text]
  2. Panagiotopoulou, P, Courcoutsakis, N, Tentes, A et al. (2012) CT imaging of peritoneal carcinomatosis with surgical correlation: a pictorial review. Insights Imaging 12, 168. doi: 10.1186/s13244-021-01110-6
